# Cases of Fractures

**Published:** 1856-12

**Authors:** Frank Hastings Hamilton

**Affiliations:** Buffalo, N. Y.


// BUFFALO MEDICAL JOURNAL

AND

MONTHLY REVIEW.

VOL. 12. DECEMBER, 1856. NO. 7.

### ORIGINAL COMMUNICATIONS.

---

ART. I.—*Cases of Fractures.* Reported by FRANK HASTINGS HAMILTON, M. D., Buffalo, N. Y.

(Continued from Page 339.)

FRACTURES OF THE FEMUR.—(CONTINUED.)—MIDDLE THIRD.

CASE XLII.—*Simple fracture. Result perfect.*

—— Rohan, an Irish lad, of Buffalo, N. Y., æt. 9, fell, July 12, 1851, and broke his left femur about its middle.

I dressed the limb with my own straight splint, using extension and counter-extension: the extension being made with a paste gaitre. I also applied side splints.

Aug. 9th, four weeks from the time of the fracture, union was complete, and without shortening or deformity. I removed the long splint, but continued the side splints.

At the end of three months the limb was in all respects perfect.

CASE XLIII.—*Simple fracture. Result perfect.*

John Gortz, of Buffalo, æt. 13, fractured his — femur in its middle third, Feb. 21, 1849. On the same day he was received into the Buffalo Hospital, and we applied a starch bandage from the knee to the toes.

Feb. 24, the bandages being dry, the straight splint was adjusted, and extension made. Lateral dressing was also applied.

March 14, the fracture appeared firm.

March 23, the bandages and splints were removed, and the leg was found sound and perfect. No excoriations had occurred on the foot or leg.

CASE XLIV.—*Simple fracture. Result perfect.*

Wm. C——, æt. 9, was kicked by a horse in the fall of 1852, in consequence of which his right thigh was broken about its middle. The fracture was simple. Dr. Cook, of Pendleton, N. Y., had charge of the limb.

Oct. [illegible]0, 1853, one year after the accident, I found the thigh not shortened, but the bone was bent forward and a little outward, at the seat of fracture.

CASE XLV.—*Simple fracture. Result perfect.*

Samuel Benson, æt. 3. Simple fracture of the thigh near its middle. A surgeon in Johnstown, N. Y., was employed.

Sixty-three years after I find the limb in every respect as perfect as the other.

CASE XLVI.—*Simple fracture. Result perfect.*

Peter John, æt. 15. Simple fracture of the femur near its middle. The fracture was treated by a surgeon in Antwerp, Belgium.

Thirty years after I could not find any traces of the accident.

CASE XLVII.—*Simple fracture. Result perfect.*

—— Madison, of Buffalo, æt. 3. May 2, 1849, he broke his femur near its middle. Dr. Sprague, of Buffalo, was called, and on the same day I met him in consultation. We dressed the limb with a roller carefully applied from the toes upward, and laid it over a double inclined plane made of pillows.

One year from this date I found the limb sound and perfect.

CASE XLVIII. — *Simple fracture. Exact result unknown.*

Isaac Fridenburgh, æt. 5, was hanging on to the back of a wagon, July 3, 1845, when his right leg caught in the wheel and broke the femur a little below its middle.

I found the fracture nearly transverse. I applied a roller to the foot and leg, and a scultetus bandage to the thigh, employing a sock gaitre for extension. The limb was secured in place by my long straight splint.

The next day his family physician, Dr. Winne, took charge of him, and I only know of the result that he recovered with a useful limb, and that he walks without any halt.

CASE XLIX. — *Simple fracture. Result imperfect.*

E. S. King, of Buffalo, æt. 19, was thrown from a sleigh Dec. 5, 1854, breaking his left femur a little below its middle. Fracture simple and oblique. By request of his family physician, I took charge of the case.

Assisted by Dr. John Boardman, I applied a straight splint, with adhesive plaster extension, and with side splints.

Jan. 6, 1855, five weeks from the time of fracture, I removed the long splint. The fragments were united, and with some overlapping, but the line of the bone seemed perfectly straight; the bone could at this time be distinctly felt.

On the 15th, six weeks after the fracture, Dr. Howell being present, it was again noticed that the line of the bone seemed perfectly straight, although the limb was shortened half or three quarters of an inch. Again on the 25th Dr. Nelson and myself made the same observation.

About this time he left his bed, and one year after I found the limb considerably bent outward at the seat of fracture.

Whether this limb was so much bent when I examined it on the 15th and 25th, and the bending could not be traced owing to the presence of some swelling, and temporary callus, or whether it occurred when he began to walk, I cannot say: I think, however, there was too little swelling immediately after the dressings were removed to have concealed the present deformity: indeed, both Dr. Nelson and myself feel quite certain that the bending must have taken place subsequently, but I do not feel warranted in affirming positively that such was the fact.

CASE L.—*Simple fracture. Result imperfect.*

Joseph Gallmeyer, æt. 9. Aug. 15, 1854, he fell from a tree, a height of about eight feet, breaking his right femur. The blow was received upon the outside of the femur. Fracture simple. The hospital record does not show in what part of the limb the fracture occurred, but I think it was in the middle third.

He was carried immediately to the Buffalo Hospital. Dr. E. P. Smith in attendance. A modification of Gibson's splint was employed.

Oct. 1, forty-six days after the accident occurred, I found the bone united, and shortened half an inch. We removed all dressings immediately, and on the 6th of Oct. he left the hospital.

CASE LI.—*Simple fracture. Result imperfect.*

Samuel Logan, æt. 18, was struck by the boom of a vessel, breaking the left femur near its middle. Fracture simple.

He was carried to the Marine Hospital, at Buffalo, on the day following, Nov. 14, 1854. The limb was dressed by Dr. Charles Wilcox, marine surgeon, and myself. We applied a straight splint, using adhesive plaster for extension. No side splints for several days. On the twenty-third day the fragments were partially united.

Dec. 13. Bony union complete. Limb shortened three quarters of an inch. Does not limp at all.

CASE LII.—*Simple fracture. Result imperfect.*

George Hanson, of Buffalo, æt. 10. George was thrown down on the sidewalk by another boy, the boy falling also upon his thigh, and breaking it near the lower end of the middle third.

Assisted by Dr. Forbush, who was at first called, but who had requested me to take charge of the case, I applied my own straight splint, with suitable side splints, &c. We used adhesive plaster for making extension.

Four weeks after I found the bone united, and shortened half an inch: the overlapping of the fragments being very perceptible to the touch. The limb was straight.

April 14th, 1853, six weeks after it was broken, he walked on the leg, and on the first of May I saw him walking with only a slight halt.

CASE LIII.—*Simple fracture. Result imperfect.*

Philip Kneck, æt 12. Simple fracture of the left femur a little above its middle, occasioned by the fall of some heavy boards upon his thigh, Sept. 13, 1855.

I applied immediately my own straight splint, with side splints, using adhesive plaster for extension. At the end of five weeks I found the bone firmly united and straight, but with an overlapping of half an inch. He does not limp.

CASE LIV.—*Simple fracture. Result imperfect.*

Frederick Frank, æt. 11. Fracture of the right femur near its middle. Simple. He was admitted into the Buffalo Hospital, April 16, 1853, eleven weeks after the fracture had occurred.

Dr. Jeyte, of Buffalo, had taken charge of the limb, and had employed in its treatment a straight splint. The bone was firmly united and straight, but it was shortened half an inch.

CASE LV.—*Simple fracture. Result imperfect.*

James Farland, æt. 6. James fell from a loaded wagon, Sept. 18, 1853, and after reaching the ground a stick of wood fell upon his right thigh, breaking it below the middle. He did not come under my care until Oct. 1st, thirteen days after the accident. I found the thigh then dressed with Gibson's splint, shortened three quarters of an inch, and not united.

Oct. 2, I substituted my own splint for Gibson's, and made extension and counter-extension with a perineal band and adhesive plasters, using also side splints, rollers, junks, &c., &c.

Oct. 18, I found the bone united.

Oct. 26, I removed all dressings. Limb shortened one quarter of an inch, and slightly bent forward and outward at the point of fracture.

After a few days he was dismissed from the hospital, cured.

CASE LVI.—*Simple fracture. Result imperfect.*

Pat Mahoney, æt. 22. Simple fracture near the middle of the right thigh. Dr. Pratt, of Buffalo, employed.

Nov. 1, 1852, eight weeks after the fracture had occurred, I found the fragments united, with a shortening of two inches. The limb was also much bent outward at the seat of fracture.

Case LVII. — *Simple fracture. Result imperfect.*

Francis Brodie, æt. 36. Admitted to the Buffalo Hospital, Jan. 5, 1853. Four months before admission his thigh had been broken near its middle. Dr. Jackson was employed as surgeon. It was treated with a straight splint. Brodie says he was visited by Dr. Jackson six times.

The limb is straight, but shortened, by overlapping, one inch.

Case LVIII. — *Simple fracture. Result imperfect.*

Graves Simcoe Lee, of Westminster, C. W., æt. 22, fell in wrestling, and fractured his right femur through its middle third, June 12th, 1851. Dr. Mixer, of Buffalo, was called, and on the following day I was associated with him. We dressed the limb with a straight and side splints, making extension by means of a paste gaitre, and the worm screw.

The fragments were firmly united in about four weeks, and with a shortening of half an inch.

Case LIX. — *Simple fracture. Result imperfect.*

Ernest Siffert, of Buffalo, æt. 8, fractured his left femur near its middle. Dr. Alden Sprague, of Buffalo, treated it with a double inclined plane.

Five years after the accident occurred, I found it shortened half an inch.

Case LX. — *Simple fracture. Result imperfect.*

Isaac Smith, æt. 5. I saw this lad but once, Aug. 29th, 1855. He was then lying in a canal boat. His left thigh had been broken eleven days by a fall from a horse. The fracture was simple and near the middle of the bone. It had been dressed with four short, side splints, by Dr. Green, of Little Falls. Every thing was now loose. The limb was shortened three quarters of an inch, and slightly bent. The toes were everted. The fragments were so firmly united that I could not, with a moderate application of force, move them.

Assisted by Dr. Boardman, I applied a double straight splint (Hagedorne's.) I could not lengthen the limb. I have never seen it since.

Case LXI. — *Simple fracture. Result imperfect.*

G. W. Hayward, of Pittsburg, Pa., while intoxicated, was thrown from a loaded wagon, breaking his left femur near its middle. Fracture simple and oblique. This occurred June 21st, 1849, and on the same day he was carried to the Buffalo Hospital of the Sisters of Charity. We immediately applied the starch bandage to his leg and foot, and seven days later we commenced extension with the straight splint.

The usual lateral splints were applied to the thigh; starch was also used freely in the dressings about this part of the limb, so that the bandages held firmly to the limb, the splints to the bandage, and the roller to the splints, and the dressings did not require re-adjustment for three weeks.

Three weeks after the fracture occurred the dressings and extending apparatus were all removed. Bone united; shortened half an inch; straight. No ulceration of heel or instep.

Limb redressed with double inclined plane. This man finally left abruptly, without settling his accounts with any of us, and I had no opportunity of again measuring the limb.

Case LXII. — *Simple fracture. Result imperfect.*

Almon Lathrop, of New York, æt. 22, broke his right femur near its middle. Simple.

He was treated by the surgeons of the New York City Hospital, with a straight splint and adhesive plaster extension.

I examined the limb, June 30, 1852, nine weeks after the fracture had occurred. The fragments were then united and the limb was shortened three quarters of an inch.

Case LXIII. — *Simple fracture. Result imperfect.*

A. Scheneiderbunger, of Baiarn, Germany, æt. 13, fractured his — thigh near its middle. The fracture was simple. A surgeon in Baiarn took charge of the limb.

Nineteen years after the accident, he was an inmate of the Buffalo Hospital. He walked without the slightest halt, and assured us that the limb was not shortened; but on measurement, I found it shortened one inch and a quarter. Drs. Vandeventer and Stewart, with myself, repeated the measurement several times, and always with the same result. The lower part of the spinal column was tilted to one side.

Case LXIV.—*Simple fracture. Result imperfect.*

Michael Hallis, æt. 15, fractured his left thigh near its middle. Fracture oblique and simple. Dr. Vaname, of Pittstown, in attendance.

Sept. 14, 1851, I examined the limb, nineteen years after the accident. I found the thigh shortened half an inch, and the hip of the same side lame.

Case LXV.—*Simple fracture. Result imperfect.*

Hiram Clark, fractured his left femur when 16 years old, near its middle. A surgeon residing at Little Falls, N. Y., was employed.

I examined the limb twenty-five years after the accident occurred, and found it shortened three quarters of an inch. He did not limp in the least when he walked, and was uncertain, until my measurement, repeated three or four times, had determined it, which leg was broken.

Case LXVI.—*Simple fracture. Result imperfect.*

John Rayner, aged about 30 years, had his left thigh broken near its middle, while serving as a United States soldier. It was treated by an army surgeon. Five years after I found the thigh shortened one and a half inches.

Case LXVII.—*Simple fracture. Result imperfect.*

Michael Heindel, æt. 25, fell from a railroad car when it was in motion, breaking his left femur a little above its middle.

Dr. Hauenstein, of Buffalo, had charge of the limb during the first fortnight, and subsequently both Drs. Sprague and Hauenstein were in attendance. They applied a straight splint, and this was removed after about five or six weeks. As soon as the splints were removed, his limb began to swell, and to become more painful. The knee was especially painful and stiff.

Seventeen weeks after the accident he called upon me, complaining of his surgeons, and asking whether the condition of his limb could be improved.

The knee was partially anchylosed. The thigh was shortened three quarters of an inch, the upper fragment riding upon the inside of the lower.

I recommended to him free motion and use of the limb, predicting that he would probably be able to walk without any lameness within six months or a year from that time.

Case LXVIII. — *Simple fracture. Result imperfect.*

Feb. 13, 1851, David Consaul, of Buffalo, æt. 30, fell about seventeen feet and struck upon his side, breaking his left femur near the middle.

Drs. Nott and Wallace, of Buffalo, in attendance. During the first twelve days the limb was treated without splints, being only supported with pillows, bolsters, &c. The thigh, and especially the knee, were very much swollen.

On the twelfth day the limb was laid over a double inclined plane made of boards, and movable at the knee joint, to which also a foot board was attached. The foot was secured to the foot board and the thigh enclosed in side splints.

These dressings were continued nine weeks and four days, being daily seen by one or the other of the surgeons, and occasionally renewed.

When the splints were finally removed, Mr. Consaul noticed a callus.

One year after Mr. Consaul called upon me to obtain advice as to the propriety of his prosecuting his surgeons.

I found the limb shortened three quarters of an inch, and somewhat bent outward at the seat of fracture. The leg is occasionally painful and not as strong as before. His knee especially "gives out" when he walks. A suit was commenced, but never brought to trial, his counsel having become convinced of its injustice, and of the impossibility of sustaining a claim for damages.

Case LXIX. — *Simple, nearly transverse fracture. Result imperfect. Prosecution and verdict for defendant.*

In Dec., 1843, William Tims, a house joiner, was employed shingling the roof of a railroad depôt, when he fell and fractured the right femur about its middle. Tims was about 40 years old. The fracture was nearly transverse.

Dr. James P. White, of Buffalo, was called, and also at the same time, Dr. Sprague. Dr. White claimed the patient and took charge of him. Assisted by Mr. Neuman, his student, he applied Sir Astley Cooper's double inclined plane, having previously covered the leg with a roller, and secured lateral splints to the thigh.

At the end of six weeks the dressings were finally removed, the fragments being united firmly.

Subsequently Tims claimed damages for malpractice in the treatment of the leg, and Dr. white was sued and the case was tried in the Erie County

Supreme Court, first in 1844 and again in June, 1845. In neither of these trials were the jury able to agree. Finally, in June, 1848, it was tried in the same court, Justice James G. Hoyt, presiding. Eli Cook, for plaintiff; J. G. Masten, S. G. Haven and James Shelden, for defence.

The limb is now shortened one inch and considerably bent or bowed forward at the seat of fracture. Drs. Trowbridge, Barnes and Burwell, witnesses on the part of the prosecution, thought it a "medium cure." Dr. Sprague did not think so. Dr. Flint, on the part of the defence, thought it an average cure. I thought it nearly, but not quite, an average cure.

The plaintiff claimed that the bend and shortening resulted from the use of a double inclined plane, and from negligence in its use; and that it occurred while the limb was in the splint. The defendant replying that it came out of the splint straight, and that the deformity now present took place after his responsibility had ceased.

Dr. Trowbridge said that "more or less deformity usually follows a fracture of the thigh bone, even in the best cases."

Dr. B. Burwell said "it was a difficult bone to heal, and to make straight."

Dr. Austin Flint said "fracture of the thigh bone is one of the most difficult to treat, perhaps the most so."

Dr. Willard Parker said "in children you may generally get union without shortening: in a well, active man you may not: it depends upon the power of the muscles."

The cause was submitted to the jury, June 30th, and the Judge charged the jury, as to the points of law, stating that if they were satisfied that the defendant had exercised ordinary skill, and ordinary care, they were to find for him. He then went into a general review of the testimony; stating also that in cases like these the medical testimony should have more weight in matters of opinion, than the testimony of other witnesses, as they were, it is to be supposed, better informed upon subjects of the nature here presented.

The jury conferred with each other a few minutes and returned a verdict for the defendant.

Case LXX.—*Simple fracture. Consolidation of the callus delayed. Result imperfect.*

Charles Henry Martin, æt. 8, fell and broke his left femur, Dec. 26, 1851. Dr. Nott was called and dressed the limb with Desault's splint. On the third day after the accident I was requested to visit the patient with Dr. Nott.

We dressed the limb carefully with side splints, and with Desault's straight splint, making all proper extension.

He was left in charge of Dr. Nott, and the long splint was continued six weeks, but he did not begin to walk, with crutches, until six weeks later. He used crutches about three or four weeks, and then for the first time walked by bearing his whole weight upon the limb. The father says he limped from the time he began to walk, and that there was a large "callus" about the fracture.

Six months after it was broken, and full four months after he began to walk, it was noticed that the limb was bending, and on the 22d of August, 1852, when the child was brought to me, the thigh was very much bent outward at the seat of fracture. There was no pain or soreness at this point, and his health was good. The father says that the change in the form of the limb was such that he could notice it from week to week; that this change seemed to him to have been going on about six weeks, and that he knows no cause for it.

I advised a resort to splints, but the patient refused absolutely to have them applied. Three months from the date of this examination the lad was brought to me again, (Dec. 1, 1852.) No treatment had been adopted. The boy had continued to run about as usual. The bending had ceased spontaneously, and indeed the bone seemed to have become a little straighter.

Case LXXI.—*Simple fracture. Broken twice in the same place, at different periods. Result imperfect.*

John Gordon, of Rochester, æt. 8, broke his thigh near its middle. Dr. Day, of Rochester, took charge of the limb.

It was again broken when he was 14 years old, at the same point. Dr. Toby, of Rochester, was employed. Both surgeons used the straight splint.

Sept. 1851, sixteen years after the last fracture, I found the limb shortened half an inch.

Case LXXII.—*Simple fracture. Union delayed. Result imperfect.*

Jacob Turinger, of Lancaster, N. Y., æt. 14, fell from a tree breaking his left thigh near its middle.

Dr. S. Potter, of Lancaster, was employed. He applied a straight splint, with extension and counter-extension. He was unable to prevent the fragments from overlapping by any amount of extension which he could employ, and as the sharp point of the upper fragment projected considerably, he did not for some time dare to apply lateral splints, and to this fact Dr. Potter attributes the delay in union.

Eleven weeks after the fracture had occurred, Dec. 13, 1848, he was admitted to the Buffalo Hospital. The fragments were not united and the limb was shortened three quarters of an inch. He says they have been united imperfectly, and that the re-fracture was produced by sitting on the edge of the bed.

I applied my own straight splint, with also side splints. Fourteen days from this time, the union of the fragments was accomplished, and as the foot had appeared swollen for some days, I removed the straight splint and continued only the side splints.

Jan. 3, all dressings were removed, and on the 10th he was discharged with the limb straight, but shortened three quarters of an inch.

Case LXXIII.—*Simple fracture. Consolidation of callus delayed. Bone accidentally re-fractured. Result imperfect.*

Joseph McEllicott, æt. 22, was crushed under a bank of earth, Oct. 27th, 1850, breaking his right femur near its middle, also his lower jaw and both bones of his left leg. The fracture of the thigh was simple and oblique.

Dr. Johnstone, of Waverly, was employed. He applied two long splints, &c.

Jan. 28, three months after the fracture occurred, he was admitted into the Buffalo Hospital. The right thigh was united, but very much bent at the seat of fracture, and shortened three inches or more. There was an ulcer under the heel of the right foot. The whole limb was swollen. An abscess existed in the left leg and an ulcer under the left heel. Patient was quite feeble. Before the accident he had enjoyed excellent health with the exception that he had, at the time of the fracture, intermittent fever.

I laid a bladder filled with water, under each heel, and suspended the limbs in a swinging fracture apparatus. The legs were ordered to be bathed in cold water twice daily.

On the 13th of February, phlegmonous erysipelas attacked the left leg, which was arrested under a tonic and stimulating treatment, after several days.

On the morning of the 17th of Feb. I found the fragments again separated: they having been refractured by an attempt to turn in bed. On the 19th I applied a starch bandage and straight splint to the right leg and thigh.

On the 24th I noticed that the limb seemed straight. March 29th I removed the dressings and found the limb united; it was now nearly six weeks since it had been re-fractured, and five months since it was first broken.

After being confined in the hospital several months longer, he began to walk, and finally recovered with a shortening in his right thigh of three inches. At no time while he was an inmate of the hospital could we extend the limb beyond this.

Cases LXXIV and LXXV.—*Simple fracture in left Femur and comminuted in right. Union delayed in latter case. Result imperfect.*

Joseph Powers, of Clyde, N. Y., æt. 23, was at work in a steam mill connected with a saw mill, when he was caught by the pump belt and drawn over the main shaft. The belt falling over his shoulders and neck lashed him fast, and carried him over, it is estimated, one hundred and fifty times, the shaft making this number of revolutions in a minute. At each revolution he passed within one foot of the ground.

The left femur was broken near its middle. The right femur was broken near its middle and in its lower third. The left humerus was also broken and the radius dislocated forward at the elbow.

Dr. ———, of Clyde, was in attendance. The radius was reduced immediately, and the humerus dressed with suitable splints. The patient was conscious, but the muscles offered no resistance, being completely paralyzed by the injury; and by the application of only moderate force, the broken femurs were brought into exact position. The severity of the injury, however, was such as at first scarcely to encourage a hope that the patient would recover, and no splints or other mechanical appliances were employed upon the lower extremities during several days. They were subsequently secured to a double inclined plane, but it was now found impossible again to reduce the fragments.

Four months after the accident I was called to see him. The left femur had then united, but it was shortened two inches, and bent forward at the seat of fracture. The right femur had united in its lower third, but no union had occurred in its middle. I rubbed the ends violently together, and then, having secured the limb in a straight splint with as much extension as he could bear safely, with also side splints snugly laid against the limb, I left him in charge of his surgeon.

I observed that no amount of extension would enable us for one moment to extend the limb more than half an inch, and that this could not be retained by any force which might be safely continued.

Three months later Mr. Powers called upon me at my office, bony union having been completed within one month from the time I saw him. He was now walking without aids, and his height was two inches less than before the accident.

Case LXXVI and LXXVII.—*Simple fracture in left Femur, and comminuted fracture in right. Limbs crooked soon after recovery, but eventually becoming straight.*

Hume, æt. 4, of Buffalo. Both of her thighs were broken April 8, 1847, by the passing over them of a wagon loaded with a cord of wood. The left femur was broken a little above its middle, and the right femur was broken at the same point, and also lower down, but in the middle third. Considerable swelling immediately followed the accident.

I dressed the legs with side splints, and laid them over a double inclined plane of boards, covered with a cotton mattress. During the whole treatment I experienced great difficulty in retaining the limbs in place, and when, at the end of three weeks, union had occurred, I found both thighs were bent forward at the seat of fracture. Whether they were overlapped or not it was impossible to ascertain.

Five years from this time the seat of fracture could not be felt, and both thighs were straight or of a natural form. The straightening had occurred spontaneously.

Case LXXVIII.—*Simple fracture. Preceded by mollities ossium. Result imperfect.*

A. M. Bartholomew, of Buffalo, æt. 41; until 31 years old enjoying good health, and with no scrophulous or other constitutional taint.

Mr. B. is a coppersmith and stands most of his time and is accustomed to lift weights considerably. During the last ten years he has not been so well. His strength and appetite having in some measure failed. He feels especially weak after dinner. A year before the fracture he noticed that his right leg was especially weak, and that the thigh was becoming bent. In 1844, when 29 years old, a large dog ran against this thigh breaking it instantly. Dr. Terry treated the fracture, employing a straight splint, without side splints. In seven weeks union was complete, but it was several months before he was able to use the limb much.

Nineteen months after this fracture, Bartholomew consulted me in relation to a bending of his left leg, which had been progressing three or four years. I found it much bent, weak, tender but not painful. The right leg had not bent any since the fracture. It was shortened one inch, and it was much the best leg of the two.

CASE LXXIX.—*Simple fracture, complicated with fracture of the patella in the same limb. Result imperfect.*

Wm. Palmerston, of Black Rock, Erie Co., N. Y., aged about 25 years, fell, Dec. 27, 1853, and broke his left thigh near the upper end of its middle third. The patella of the same leg was broken at the same time. Dr. Lewis, of Black Rock, was in attendance. I saw the patient with Dr. Lewis, on the fifth day.

For the fracture of the femur we used a single long straight splint, with adhesive plaster extension, and side splints.

The femur has united with a shortening of half an inch, (see fractures of the patella.)

CASE LXXX.—*Simple, oblique fracture in a paralyzed limb. Result probably perfect, but somewhat uncertain.*

Willis Sawens, of Albion, Orleans Co., N. Y., æt. 14, broke his right femur near its middle, by a fall from a horse. Direction of fracture obliquely downward and outward.

When only eight months old, after a severe illness, this limb had been weak, and had become gradually much emaciated. It was also two or three inches shorter than the other at the time of the fracture.

Within three or four hours after the accident, before the limb was much swollen, it was reduced by Dr. Hughes, of Paris Centre. Very little swelling ever occurred. Two men reduced it, using, as Mr. Sawen thinks, considerable force. The fracture was dressed with a roller applied the whole length of the limb, and a side splint, of about seven inches in length, made of basswood, which having been planed sufficiently thin, was steamed and bent around the leg so as to fit accurately. Between the splint and roller was placed a wide compress. The splint was secured with strings. The limb was then laid over a double inclined plane made of pillows.

He was kept in bed three months, and the lateral splint was not removed until after four months and a half.

No contractions or spasms occurred in the leg during the treatment, and his health continued constantly quite good.

Eight years after the limb was examined by Prof. Charles A. Lee and myself. We found it about two and a half inches shorter, and in circumference about one-third as large as the other. The muscles were soft and flabby. We could not, however, discover the seat of fracture, and Mr. Sawens, then a student of medicine, assured us that the limb was as long as before.

CASE LXXXI.— *Compound fracture. Result imperfect.*

John Street, of Buffalo, æt. 18, was injured by the fall of a bar of iron upon his right thigh, breaking it near its middle. One of the fragments was pushed through the skin.

On the same day he was admitted to the Hospital of the Sisters of Charity. I applied a Potter's straight splint and made extension with adhesive straps, &c.

The wound in the flesh healed promptly, and the bone united in the usual time. The extension was discontinued at the end of six weeks.

He left the hospital ten weeks after admission with a shortening of the thigh of one inch and a quarter. The limb was strong and the halt scarcely perceptible. I think the unusual shortening was due to the fact that the bone had penetrated, and by wounding the muscles, had caused them to contract.

CASE LXXXII.— *Simple fracture. Result imperfect.*

John Laughton, æt. 7, admitted to the Buffalo Hospital, Jan. 26, 1853, with a fracture of the femur near its middle. He had fallen, January 12th, from a ladder, a height of nine feet. Dr. Wyckoff, of Buffalo, dressed the limb with Gibson's splint.

I substituted, for Gibson's splint, my own, and covered the foot and leg with a paste bandage, making extension with adhesive straps.

On the thirty-ninth day the union was firm, and I removed all dressings on the forty-fifth day. The limb was then shortened half an inch, but straight. The overlapping of the fragments felt like ensheathing callus.

CASE LXXXIII.— *Compound fracture from a gunshot wound. Result nearly perfect.*

A. B., æt. 24. This gentleman was shot, June 7th, 1855, through both thighs. The bullet, discharged at ten paces from a large dueling pistol, entered the right thigh eight inches above the knee, passing a little obliquely downward and inward, in front of the bone, and then entering the left thigh perhaps one inch lower down and half an inch farther back so as to strike and fracture the left femur near the upper end of its lower third. The bullet buried itself in the left thigh and has never been found. A portion of the pantaloons and drawers entered with the bullet and were drawn out before my arrival. Rollers were applied moderately firm, and cold water dressings directed; absolute rest was also enjoined.

This plan was pursued until the eighth day after the fracture. The bullet had passed in such close proximity to the femoral artery in both thighs, that it was thought most prudent, so long as there was no muscular spasms and very little displacement, to withhold the application of splints. Indeed we were in constant apprehension that a dangerous hæmorrhage would occur.

On the second day the thigh and leg of the broken limb were very much swollen; he was suffering with a dull, heavy pain in his thigh, and especially behind the knee; his sleep was disturbed; his pulse 120, and wiry.

These symptoms gradually disappeared, and on the eighth day, June 15, I applied my own straight splint, with adhesive plaster extension. At this time the swelling had greatly subsided, but there was extensive discoloration of the limb from bloody effusion. The wounds were beginning to suppurate.

Aug. 1, eight weeks after the fracture, we removed the large splint and found the bone united with the line of the limb perfect, but with an overlapping of half an inch. He remained in bed one week longer, when, the wounds having been for some time completely closed, he was permitted to get upon crutches.

He now walks without the smallest amount of halting, and the limb is in all respects as useful as before. This result, so unexpected in a case where so many complications were presented, was due in no small degree to the remarkable firmness and composure of the patient, as well as to the care and skill of Dr. Ware, of Niagara Falls, and Dr. Church, of New York, the first of whom was in constant attendance throughout the whole course of the treatment.

Case LXXXIV.— *Comminuted fracture. Result imperfect.*

John Rayner, a discharged Florida soldier, aged about 35 years, was lying upon his back between the rails of the Syracuse and Auburn railroad, when the train passed over him, the fire pan striking the knee of his right leg as it was drawn up, and breaking it in two places: one fracture being through the middle and one through the lower third of the femur.

I dressed the fracture on the same day, with side splints and Bowen's double inclined plane, called also the "navy" splint. The left thigh had been broken in Florida and had united with a shortening of about one and a half inches. (See Case LXV.)

The accident occurred in the fall of 1839, and union took place in the usual time, but with a shortening of about two inches, and the fragments remaining slightly bent forward at the seat of fracture. This shortening and bend I found it impossible to prevent, the limb being greatly swollen for many days, and not admitting of any degree of extension.

Case LXXXV.— *Compound, comminuted fracture. Result imperfect.*

J. A. Burrows, æt. 14, fell fifty feet, striking upon his knee and fracturing his left femur through its middle. Fracture compound and comminuted.

Dr. David Durfee, of Penfield, Monroe Co., N. Y., was employed, and laid the limb upon a double inclined plane.

He did not use the leg for more than a year. About eighteen months after it had been broken, a small piece of bone escaped through a fistulous opening. Seventeen years after the accident, while he was a student of medicine, I examined the thigh and found it firm, but shortened one inch. The whole limb is atrophied, but he walks without scarcely any limping.

Case LXXXVI.— *Compound, comminuted fracture. (Gunshot fracture.) Result imperfect.*

Pat Haney, of Livingston Co., N. Y., æt. 23, had his thigh broken near its middle, by a bullet, while he was serving as a private soldier in the English army. He was treated by an army surgeon.

Thirty-eight years after, when I saw him in the Livingston County Alms House, N. Y., the thigh was shortened one inch and a quarter, but the limb was quite useful.

Case LXXXVII.— *Compound fracture, complicated with a fracture of the tibia and fibula. Death from the shock in fifty-four hours.*

Catherine Henriger, of Buffalo, æt. 68, jumped from a second story window, March 15, 1853, producing a compound fracture of the left femur, near its middle, and also an oblique fracture of the tibia and fibula of the right leg, at the lower end of the upper third. She was not brought to the hospital until the following day.

We applied splints to steady and support the limbs, and finding her sinking, we endeavored to sustain her with stimulants, &c.; she continued to fail, however, and died fifty-four hours after the injury was received.

### LOWER THIRD.

#### REMOTE FROM CONDYLES.

Case LXXXVIII. — *Simple fracture. Result perfect.*

James Bryan, of Buffalo, æt. 8, fractured his — femur, at the upper end of the lower third, Jan. 20, 1849. Fracture simple. He was admitted to the Buffalo Hospital on the next day.

Jan. 23d, we applied the starch bandage to his foot and leg.

Jan. 28th, seven days after the occurrence of the fracture, extension and counter-extension were made with a straight splint, and lateral splints were also applied.

This treatment was continued until the seventeenth of February, a period of twenty days, and extending to twenty-seven days after the fracture, during which time the little patient made no complaint, but was always cheerful and happy.

On the 17th of February, the bandages and splints were removed. The bone had united with proper length and direction. There was not even discoloration, or tenderness, about the ankle or leg.

Case LXXXIX. — *Simple, transverse fracture. Result perfect.*

Aug. 13, 1847, Harris, a lad 3 years old, fell from a ladder, breaking his left thigh near the upper end of the lower third. I think the fracture was transverse. Assisted by Dr. James E. King, of Buffalo, I dressed the limb a few hours after the accident.

We applied lateral splints, made of felt, and then laid the whole limb over a double inclined plane.

On the fifth day I re-dressed the fracture and found every thing in place.

On the thirteenth day the limb was bent and overlapped. Finding that the fragments had not united, I immediately substituted a simple straight splint, such as I usually employ for children, making, at the same time, as much extension as he would bear.

One year after, the limb was straight, nor was there any perceptible shortening.

CASE XC. — *Simple fracture. Result imperfect.*

Abram Miller, of Steuben Co., æt. 24, fractured his right femur through its lower third. Fracture simple.

Dr. White, of Wyoming, N. Y., was employed. The limb was dressed with side splints and laid over a double inclined plane.

Thirty years after the accident, I found the thigh shortened one inch and three quarters; yet he walked with only a very slight halt.

CASE XCI. — *Simple fracture. Result imperfect.*

Elisha Standish, æt. 42, fractured his thigh through its lower third. A surgeon in Livingston Co., N. Y., whose name he has forgotten, dressed and took charge of the limb.

Five years after the accident had occurred, I found him an inmate of the Livingston County Alms House. His thigh was shortened one inch.

CASE XCII. — *Simple fracture. Result imperfect.*

H. Hogan, of New York, æt. 42, fractured his — femur through its lower third.

It was treated at Bellevue Hospital, N. Y., with a straight splint, and with adhesive plaster extension.

I examined the limb seven weeks after the fracture had occurred, and found it shortened half an inch.

CASE XCIII. — *Simple fracture. Result imperfect.*

Henry Dugan, of Buffalo, æt. 11. Fracture of right femur about three or four inches above the knee joint. Simple. Occasioned by being tripped in wrestling, and falling with his legs spread asunder.

The limb was immediately dressed by Drs. T. Lockwood and Charles H. Baker; and on the same or following day, Feb. 5, 1853, he was carried to the hospital. I found the limb enclosed very neatly, in long splints made of binders' board, carefully moulded to the limb and secured in place with a roller extending from the toes upward. In order that I might examine the fracture I removed these dressings. I thought the fracture was transverse. I now dressed the limb carefully myself with first a paste bandage, extending from the toes to above the knee. I then applied lateral splints to the thigh and a long splint for extension. Extension was made with adhesive straps. Great care was taken in the dressings, and when they were completed the two limbs were of the same length. I measured with a tape line.

March 5th, I removed the splint. The fragments were united, but with a shortening of three quarters of an inch. The upper fragment overlapping and lying upon the outside of the lower. This result has happened notwithstanding I took unusual care, throughout the treatment, to keep up the extension, and notwithstanding the patient was in every way submissive.

Case XCIV.—*Simple fracture. Result imperfect.*

Sarah Sullivan, æt. 27, admitted to the Buffalo Hospital, March 9, 1851, with a fracture of the right femur, near the upper end of the lower third. The limb had not been dressed.

I ordered a paste gaitre to be applied to her foot and leg, and on the 10th the house surgeon applied the long thigh splint with extension and counter-extension; also the usual lateral splints.

Union occurred in about five weeks, with a shortening of three quarters of an inch. Mrs. Sullivan was a feeble woman, and did not bear confinement well. At several times we found she had loosened her bandages herself.

Case XCV.—*Simple fracture. Result imperfect.*

Alonzo Benedict, of Buffalo, æt. 10, was run over by a wagon loaded with lumber, Sept. 24, 1852, breaking his — femur at the upper end of the lower third. I could bend the limb at the point of fracture, with great freedom, yet I could not produce crepitus. I was unable to determine at first whether it was not broken also at a point lower down. I now think it was not.

I applied my double, long splint, making extension with adhesive plasters, and confining the whole length of the limb very snugly to the long splint. At first, and for several days, I used no small side splints upon the thigh. When the dressings were applied I measured the limbs and found them of the same length.

Oct. 22, four weeks after the occurrence of the fracture, I removed the apparatus in presence of Dr. Bowen, and found the fragments united with a shortening of one quarter of an inch. There was also a slight forward projection at the seat of fracture.

Sept. 15, 1856. He walks without any halt, and has never suffered any inconvenience from the limb.

CASE XCVI.— *Simple fracture. Result imperfect.*

Lyttle, æt. 20. Femur broken four inches above the knee, obliquely, by the fall of a bank of earth.

Five hours after, when I dressed the fracture, it was much swollen. I restored the fragments, as completely as possible, with pulleys, and then applied Gibson's splint, allowing the pulleys to remain for a few days.

March, 1837, three months after the accident, he was walking without any perceptible halt. The limb was, however, shortened half an inch.

CASE XCVII.— *Comminuted fracture. Union with shortening.*

(Same as Case LXXXII, of middle third.)

CASE XCVIII.— *Simple fracture. Result imperfect.*

Feb. 28th, 1846, John Burscough, a very muscular and fat butcher, was thrown from his sleigh and broke his thigh at the upper end of the lower third. Dr. Mixer and myself in attendance.

We placed him temporarily on a double inclined plane, having no straight splint at hand. On the following day we applied the straight splint. At the end of four weeks we loosened the extension and found an ulcer on the heel. The bones were not united. At the end of six weeks we removed all extension. The bones were then united. The ulcer did not close under a month or two. The thigh was shortened half an inch.

CASE XCIX.— *Simple fracture. Union delayed. Result unknown.*

Pat McDonald, of Medina, N. Y., æt. 23. His left thigh was broken in its lower third by being traversed by a hand car.

He was at first seen by Drs. Whaley, Holt and Frost, of Medina. A splint was applied and extension employed, but on the fourth day it became so painful that the extension was discontinued. On the eighteenth day extension and counter-extension were again employed, and continued for some time.

Feb. 18, 1853, seven weeks after the fracture occurred, he was brought to the Buffalo Hospital. The fragments were still ununited. He was pale and feeble; leg and foot much swollen; excoriations about the thigh; a sloughing ulcer soon occurred upon the heel.

I did not apply any splints until March 9th, having only employed, until this time, means to re-establish his health. I then applied a paste roller and splints of binders' board. He was directed also to get up on crutches.

About one month after admission I found the bones united, and, my record says, without any shortening, but I do not know by whom the measurement and record was made.

CASE C.—*Simple fracture, complicated with a fracture of the tibia and fibula in the same leg. Result imperfect.*

Thomas Dolanty, aged about 30 years. August 14th, 1854, his leg and thigh were traversed by a loaded cart, breaking the thigh near the upper end of the lower third, and the leg about eight inches above the ankle; the latter accident was compound.

Assisted by Austin Flint, jr., I dressed both fractures carefully, applying my own long splint. Extension, so far as we were able to make it, was effected through the aid of a paste gaitre.

On the tenth day Dr. Boardman, one of the hospital surgeons, took charge of him at my request.

At the end of eight weeks the femur was united with an overlapping of three quarters of an inch. It was perfectly straight. The leg united with a shortening of one quarter of an inch.

CASE CI.—*Compound fracture. Result imperfect.*

George Foster, of Brantford, C. W., fell fifty-five feet, September 5, 1855, breaking his left thigh obliquely, near the upper end of the lower third. The fracture was compound. His elbow joint was dislocated at the same time.

Mr. Foster called upon me at Buffalo, I think in March, 1856, about six months after the accident had happened. He stated to me that he was 23 years old. That the limb was treated by Dr. ———, of Brantford, C.W.; the limb was laid over a double inclined plane, and the thigh was supported with thigh splints; no means for permanent extension were employed; the limb was kept upon the splint seven weeks; two months after it was broken and when it had united, Dr. —— applied extension, and the fracture was reproduced. The thigh was now enveloped in a mould of plaster of Paris; this was removed at the end of one week; in three weeks more the fragments were again firmly united.

When I examined the limb it was united, but with a shortening of three inches; the foot and leg were slightly rotated outward; an abundant callus surrounded the fracture, and a fistulous discharge still continued from the bone. His knee was stiff and his whole leg greatly swollen. He was unable to walk or stand.

Case CII.— *Compound, complicated fracture. Amputation. Death from the shock twenty-six hours after the accident.*

Martin, æt. 4. July 5, 1854, a locomotive on the Niagara Falls Railroad traversed his right thigh and leg. Thigh broken at the upper end of the lower third. Leg torn off.

Jan. 6th, æt. 10, A. M. Eighteen hours after the accident. Assisted by Drs. Boardman, Root and White, I amputated the thigh four inches below the trocanter major. He had never completely rallied from the shock, and he died at 6 o'clock, P. M.

Case CIII.— *Compound, comminuted fracture of both thighs. Death from the shock thirty hours after the accident.*

Hiram Birbanks, æt. 20, was crushed between a couple of boats lying in the harbor, May 1, 1851. Both thighs were broken into two or more fragments, through their lower thirds. The flesh and skin were also severely torn.

I dressed the wounds carefully, but applied no splints. Notwithstanding the administration of stimulants and opiates, he never recovered from the shock, and died thirty hours after the injury was received.

JUST ABOVE CONDYLES.

Case CIV.— *Simple fracture. Result imperfect.*

James A. Manley, æt. 4, fractured the right femur about two inches above the joint. Probably it was a separation of the epiphysis from the diaphesis.

The limb was dressed, and subsequently treated, by a surgeon living in Hubbarton, Vermont.

Twenty-six years after the accident occurred, in July, 1853, I examined the leg. The point of fracture could not be felt, yet the thigh was shortened half an inch. He walked without any halt, and he was not aware that the limb was shortened.

Cass CV.—*Simple fracture. Result imperfect.*

Wm. Hennen, æt. 25, was caught between a carriage and a tree, Feb. 13, 1854, breaking his left femur three and a half inches above the knee-joint. Dr. Layton dressed the fracture ten hours after it occurred. He applied two long splints, one on each side of the thigh and leg, with rollers, &c. Extension was made by four men, before the splints were applied, but no extension was employed afterward. The dressings were removed and the limb examined occasionally. The splints were continued six weeks.

June 21, 1854, Hennen called upon me. The limb was shortened one inch and a half or three quarters; the lower end of the upper fragment lying in front and upon the outside of the lower. He walked with a halt. Motions of knee-joint free.

His object in calling upon me was to ascertain whether the limb had been properly treated, and whether it could not be broken over again.

Case CVI.—*Simple fracture. Union delayed. Limb shortened.*

Andrew Carr, of New York, æt. 25, fractured his right femur about three inches above the joint, and was taken to the New York Hospital.

I examined his thigh, June 30, 1852, nearly five months after the fracture had occurred. The fragments were not united, and the limb was shortened three quarters of an inch. The fracture had been treated with a straight splint.

Case CVII.—*Compound, comminuted fracture. Exfoliation of bone and great shortening.*

Daniel Welch, of Ireland, æt. 20, was crushed under a heavy weight, breaking and comminuting his right femur just above the knee. Subsequently several pieces of bone came out.

June 30, 1851, five years after the accident, in presence of Drs. Pupikoffer, Boardman and Nichols, I examined the limb. Welch told us at first that the limb was shortened three inches, but on careful and repeated measurements, we found it was shortened seven inches! To compensate for all this loss in the length of the limb, he had raised the heel of his shoe one inch, and now he walked with scarcely any perceptible halt. An examination of his back, however, furnished an easy solution of this seeming paradox. His spine was very much bent to the right in the lumbar region, so that constantly the right wing of his pelvis was four inches lower than the left, and when he bore his weight upon his right leg in walking, the right wing of his pelvis was six inches lower than the left.

Case CVIII. — *Simple fracture complicated with injury of the poplitral vessels and nerves. Gangrene of foot.*

George Taylor Aikin, of Lockport, N. Y., æt. 7. May 18, 1854, in jumping down a bank of about three feet in height, he broke the right thigh obliquely, just above the knee-joint. Direction of the fracture obliquely downward and backward.

Dr. Wm. B. Gould, an accomplished surgeon, residing in Lockport, was called. The limb was not then much swollen. He applied side splints, rollers, &c., carefully, and then laid the limb over a double inclined plane, (Day's.) The knee was elevated about six or eight inches. Before applying the splints, suitable extension had been made, and after completing the dressings the two limbs seemed to be of the same length.

These dressings were examined and re-adjusted daily. On about the seventh day the lad was complaining a good deal of pain, &c. He had all along been restless and had complained, at times, of pain in his leg and foot. Dr. Gould now noticed for the first time that his toes looked unnaturally white, and that they were cold.

Counsel was now called at the request of Dr. Gould, when it was determined to abandon all dressings and direct their efforts solely to saving the limb.

The result has been that slowly a considerable portion of his foot died and sloughed away, leaving only the tarsal bones. The fracture united, but with considerable overlapping and deformity.

Feb. 26, 1856, the case was brought to me by his father. His general appearance indicated delicacy. On examining the fracture I noticed that the anterior line of the bone seemed nearly straight, only bending back slightly about three inches above the joint, and this was owing in some degree to the covering of soft parts, and in some degree, also, to the manner in which the fragments rested upon each other: the pointed superior end of the lower fragment resting snugly upon the front of the upper fragment, so that no abrupt angle existed in front. On the back of the limb, however, the lower end of the upper fragment, quite pointed, projected freely downward and backward into the popliteal space, so that its extreme point was only about half an inch above the line of the articulation. The limb had shortened one inch, and this enabled us to determine accurately that the lower point of fracture in the bone was one inch and a half above the articulation.

The motions of the knee-joint were pretty free. The leg was extremely wasted, and the anterior half of the foot having sloughed off, the sores had now completely healed over. He was able to walk tolerably well without either crutch or cane.

Subsequently, Dr. Gould found it necessary to sue the father of the child for the amount of his services, when Mr. Aikin put in a plea of malpractice, and that consequently the services were without value.

The case was tried in the March term of the Niagara circuit for 1856, at Lockport, Hon. Benj. F. Greene, presiding. As counsel on the part of the plaintiff, (Dr. Gould,) were employed John M. Talcott, of Buffalo, and Phineas L. Ely, of Lockport; and on the part of the defendant, E. J. Chase and W. S. Farnell, both of Lockport.

On the part of the defence it was claimed that the death of the foot was in consequence of the bandages being too tight. They failed, however, to show that they were extraordinarily or unduly tight. While on the part of Dr. Gould, the prosecutor, it was shown that the death of the toes was preceded by a loss of color, and that it was not accompanied with either venous or arterial congestion. The medical gentlemen examined as witnesses, declared that this circumstance furnished the most positive evidence which could be desired, that the death of the toes was not due to the tightness of the bandages, but that its cause must be looked for in an arrest of the arterial or nervous currents supplying the limb, or in both. They believed, also, that the projection of the superior fragment into the popliteal space was sufficient to cause this arrest. They also believed that this overlapping and consequent projection, could not have been prevented in this case, and that therefore the treatment was not responsible for this unfortunate result: indeed they regarded the treatment as correct, and the result as a triumph of skill, in that any portion of the limb was saved; the leg and foot now remaining being far more useful than any artificial leg and foot could be.

The Hon. Judge, in a speech remarkable for its clearness and liberality, sought to impress upon the jury the value of the medical testimony. The jury returned a verdict for Dr. Gould, allowing the amount of his claim for services, with the costs of suit.

CASE CIX.—*Fracture of femur, complicated with fracture of the leg, and with other injuries. Death on the tenth day.*

Michael O'Shea, æt. 40. A heavy timber fell upon his left leg, breaking the femur obliquely close above the condyles. The tibia was also partially displaced inward, the astragalus dislocated, and the fibula broken two inches from the ankle. He was admitted to the hospital on the same day, Dec. 12, 1852.. We found also a large lacerated wound in front of the tibia near the ankle, bleeding freely; and a still more extensive laceration of the integuments covering the upper part of the thigh, and which had opened the saphenic vein.

We dressed the wounds carefully, but applied no splints. On the third day the wounds looked gangrenous, and on the tenth day he died. The dislocation of the astragalus was not discovered until after death.

(To be Continued.)

